# The Inquisitive Antelope

**Published:** 1892-06

**Authors:** 


### THE INQUISITIVE ANTELOPE.

An antelope is as curious as a woman. If the hunter will lie down in the grass and wave a red handkerchief, a band of antelope will keep circling round until within reasonable distance for a safe shot. After completing a circle the antelope halts suddenly and brings down one fore foot with a vigorous stamp on the ground and at the same instant makes a sort of a snort that sounds like a half-whistle. This is the propitious moment for peppering them with rifle balls. I learned this trick when a frontiersman came along and found me crawling for miles on the level prairie endeavoring to get a shot at one of the timid creatures. The man asked me if I thought I could get him. I answered: "Get him! I've got to get him; I'm out o' meat." He then posted me about lying still and flirting with the handkerchief, and I found they liked that better than chasing, and I made an entry right there that an antelope possessed some of the characteristics of a woman. After that I experimented with them (the antelope) a great deal. I found that if I wanted them to go the right, the way to manage it was for me to ride to the left of a given point. The antelope would imagine that I was trying to head him off in that direction, and he would run with all his might across the course that I was taking. I would then change my course and ride to the right and the antelope would rush back in that direction, thinking I wanted to cut off his escape, on that line. By manouvering in that way, back and forth, and all the while advancing in a general direction, I have driven a band right into my camp, where the boys would shoot them down. The game would be so intent trying to outwit me that they would never see the camp until it was too late. I have ridden rapidly entirely away from a band of antelope, and their curiosity would lead them to pursue me for hours across the plain and over the hills. One moonlight night, when the mirage was very strong and magnified a band of antelope until they appeared as large as a band of horses, I knew a party of cavalrymen to fire on them under the mistaken notion that they were sending a volley among a party of hostile Indian warriors.

